# Letter to the Editor From J. Finsterer: “Explaining Long COVID: A Pioneer Cross-Sectional Study Supporting the Endocrine Hypothesis”

**DOI:** 10.1210/jendso/bvae086

**Published:** 2024-04-26

**Authors:** Josef Finsterer

**Affiliations:** Neurology Dpt., Neurology & Neurophysiology Center, 1180 Vienna, Austria

We read with interest the article from Ach et al on a cross-sectional study of corticotrophic and somatotrophic functions in 64 patients (G1: fully recovered [n = 32], G2: had long COVID [n = 32]) during a postinfection period of 3 to 15 months after the acute SARS-CoV-2 infection (SC2I) [1]. G2 had more frequent deficits in the anterior pituitary gland compared to G1 [1]. Baseline and peak cortisol and growth hormone levels were significantly lower in G2 than in G1 [1]. It was concluded that the endocrine hypothesis of anterior pituitary insufficiency can be considered as an explanation for long COVID [1]. The study is impressive, but, in addition to the limitations mentioned in the article, some points require discussion.

The first point is that the dependence of cortisol serum levels on the amount of chronic stress to which the included patients were exposed to during the study period was not reported. Therefore, we should know whether stress has been quantified using, for example, the perceived stress questionnaire (PSQ), the stress and coping inventory (SCI), the perceived stress scale (PSS), or the standard stress scale (SSS), and included in the assessment. We should also know how many of the included patients had returned to work during the study period and how many were still on sick leave.

The second point is that it remained unclear how central nervous system (CNS) impairment in the acute stage of the SC2I, particularly hypophysitis or hypothalamic lesions, could be excluded as a cause of pituitary insufficiency. Hypophysitis is known to be a CNS complication of SC2I [2]. We should know whether pituitary hormone determination and pituitary magnetic resonance imaging were performed in all enrolled patients in the acute stage of SC2I.

The third point is the discrepancy regarding the exclusion criteria. According to the methods section, any pathology that could interfere with the hormonal assessment was excluded. However, according to Table 1, several patients in G1 and G2 had diabetes. The study cohorts also included several patients with dyspnea, glycemia, and goiter. These discrepancies should be resolved.

A fourth point is that alternative hypotheses to explain long COVID and arguments against the endocrine hypothesis have not been discussed in detail. The strongest argument against the endocrine hypothesis to explain long COVID is that it may only explain some manifestations of long COVID, such as fatigue, exercise intolerance, or brain fog but not many other manifestations. Manifestations of long COVID that cannot be easily explained by the endocrine hypothesis include chronic myocarditis [3], mast cell activation syndrome (MCAS) [4], coagulopathy [5], myositis, dyspnea, arthralgia, multi-inflammatory syndrome (MIS), or dysautonomia. More plausible than the endocrine hypothesis to explain the long COVID syndrome are mechanisms such as the persistence of the virus, hypercoagulopathy, immune dysregulation, autoimmunity, hyperinflammation, or a combination thereof [6].

In summary, the excellent study has limitations that make the results difficult to interpret. Removing these limitations could strengthen the study's message. Before long COVID can be explained by the endocrine hypothesis alone, several other speculations that claim to explain the disease must be ruled out as causal.

## Abbreviation

SC2I: SARS-CoV-2 infection

## References

[1] Ach T , Ben Haj Slama N, Gorchane A, et al Explaining long COVID: a pioneer cross-sectional study supporting the endocrine hypothesis. J Endocr Soc. 2024;8(3):bvae003. 10.1210/jendso/bvae003 PMC10801829 38260089

[2] Misgar RA , Rasool A, Wani AI, Bashir MI. Central diabetes insipidus (Infundibuloneuro hypophysitis): a late complication of COVID-19 infection. J Endocrinol Invest. 2021;44(12):2855‐2856. 34215999 10.1007/s40618-021-01627-z PMC8253675

[3] McMaster MW , Dey S, Fishkin T, Wang A, Frishman WH, Aronow WS. The impact of long COVID-19 on the cardiovascular system. Cardiol Rev. 2024. doi: 10.1097/CRD.0000000000000654 38285646

[4] Sumantri S , Rengganis I. Immunological dysfunction and mast cell activation syndrome in long COVID. Asia Pac Allergy. 2023;13(1):50‐53. 37389095 10.5415/apallergy.0000000000000022 PMC10166245

[5] Baroni M , Beltrami S, Schiuma G, et al In situ endothelial SARS-CoV-2 presence and PROS1 plasma levels alteration in SARS-CoV-2-associated coagulopathies. Life (Basel). 2024;14(2):237. 38398746 10.3390/life14020237 PMC10890393

[6] Tziolos NR , Ioannou P, Baliou S, Kofteridis DP. Long COVID-19 pathophysiology: what do we know so far? Microorganisms. 2023;11(10):2458. 37894116 10.3390/microorganisms11102458 PMC10609046

